# 2-(2-Pyrid­yl)pyridinium (2,2′-bipyridine-κ^2^ *N*,*N*′)tetra­kis­(nitrato-κ^2^ *O*,*O*′)bis­muthate(III)

**DOI:** 10.1107/S160053681103769X

**Published:** 2011-09-20

**Authors:** Zhao-Hui Meng, Shu-Shen Zhang

**Affiliations:** a College of Chemistry and Pharmacy Engineering, Nanyang Normal University, Nanyang 473061, People’s Republic of China

## Abstract

The structure of the title compound, (C_10_H_9_N_2_)[Bi(NO_3_)_4_(C_10_H_8_N_2_)], consists of 2-(2-pyrid­yl)pyridinium cations and anions [Bi(NO_3_)_4_(C_10_H_8_N_2_)]^−^. The Bi^3+^ ion lies on the twofold axis. It is coordinated by two nitro­gen atoms from one 2,2′-bipyridine ligand and eight oxygen atoms from four NO_3_^−^ anions. The disordered cation is positioned at the inversion centre. The [Bi(NO_3_)_4_(C_10_H_8_N_2_)]^−^  anions and 2-(2-pyrid­yl)pyridinium cations are connected *via* N—H⋯O hydrogen bonds into chains. Moreover, these chains are further linked into a two-dimensional layered structure through π–π stacking inter­actions between bipyridine ligands along the *c* axis [centroid–centroid distance = 2.868 (4) Å].

## Related literature

For potential applications of bis­muth(III) coordination compounds in medical chemistry, see: Sun & Szeto (2003 ▶); Sun *et al.* (2004 ▶). For reported bis­muth(III) coordination compounds, see: Andrews *et al.* (2006 ▶); Boitrel *et al.* (2003 ▶); Marsh (2002 ▶); Wullens *et al.* (1998 ▶); Yang *et al.* (2006 ▶, 2007 ▶). For the structure of disordered protonated 2,2′-bipyridine, see: Bowmaker *et al.* (1998 ▶). For the bond-strength calculations, see: Brown & Altermatt (1985 ▶); Brese & O’Keeffe (1991 ▶).
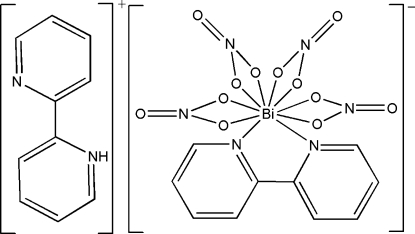


## Experimental

### 

#### Crystal data

(C_10_H_9_N_2_)[Bi(NO_3_)_4_(C_10_H_8_N_2_)]
*M* *_r_* = 770.40
Monoclinic, 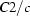

*a* = 14.711 (5) Å
*b* = 10.169 (3) Å
*c* = 16.832 (5) Å
β = 97.275 (6)°
*V* = 2497.7 (13) Å^3^
*Z* = 4
Mo *K*α radiation
μ = 7.14 mm^−1^
*T* = 293 K
0.26 × 0.24 × 0.18 mm

#### Data collection

Bruker APEXII CCD diffractometer
Absorption correction: multi-scan (*SADABS*; Bruker, 2008 ▶) *T*_min_ = 0.258, *T*_max_ = 0.360
5998 measured reflections
2224 independent reflections
1895 reflections with *I* > 2σ(*I*)
*R*_int_ = 0.036

#### Refinement

*R*[*F* ^2^ > 2σ(*F* ^2^)] = 0.035
*wR*(*F* ^2^) = 0.084
*S* = 1.02
2224 reflections
194 parameters
11 restraints
H atoms treated by a mixture of independent and constrained refinement
Δρ_max_ = 1.13 e Å^−3^
Δρ_min_ = −1.43 e Å^−3^

### 

Data collection: *APEX2* (Bruker, 2008 ▶); cell refinement: *SAINT* (Bruker, 2008 ▶); data reduction: *SAINT*; program(s) used to solve structure: *SHELXS97* (Sheldrick, 2008 ▶); program(s) used to refine structure: *SHELXL97* (Sheldrick, 2008 ▶); molecular graphics: *SHELXTL* (Bruker, 2008 ▶); software used to prepare material for publication: *SHELXTL*.

## Supplementary Material

Crystal structure: contains datablock(s) global. DOI: 10.1107/S160053681103769X/yk2019sup1.cif

Additional supplementary materials:  crystallographic information; 3D view; checkCIF report

## Figures and Tables

**Table 1:** Selected bond lengths (Å)

Bi1—N3	2.444 (5)
Bi1—O5	2.470 (6)
Bi1—O2	2.564 (6)
Bi1—O4	2.626 (6)
Bi1—O1	2.703 (8)

**Table 2:** Hydrogen-bond geometry (Å, °)

*D*—H⋯*A*	*D*—H	H⋯*A*	*D*⋯*A*	*D*—H⋯*A*
N4—H7⋯O4	0.93	2.31	3.145 (10)	149

**Fig. 1.**
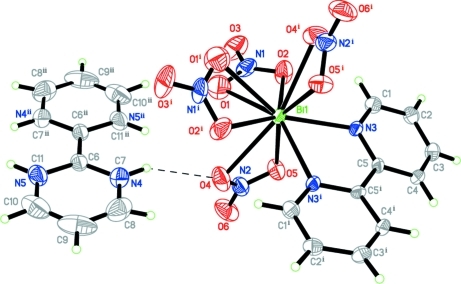
View of the title compound with displacement ellipsoids drawn at the 30% probability level (symmetry codes: (i) -x, y, -z + 1/2; (ii) -x, -y, -z).

**Fig. 2.**
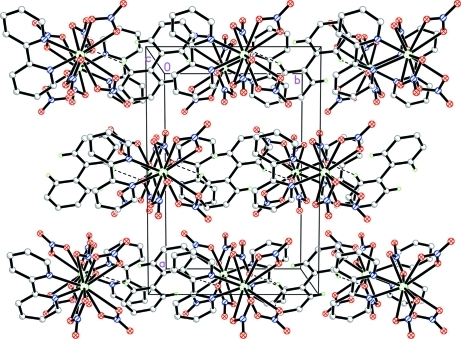
Crystal packing viewed along the c axis. For clarity, H atoms bound to C atoms are omitted, and only H atoms bound to N atoms are shown.

## supplementary crystallographic information

### Comment

The bismuth (III) coordination compounds have attracted considerable attention in recent years due to their fascinating structural architectures and potential applications in chemical industry, bioengineering, medical chemistry and so on. Up to now, a large number of bismuth (III) coordination compounds with various structural motifs and dimensionalities have been reported. As an expansion of such class of compounds, we have successfully isolated a novel bismuth (III) compound (C_10_H_9_N_2_)[Bi(C_10_H_8_N_2_)(NO_3_)_4_] which exhibits a two-dimensional layered structure built up from monoprotonated 2,2-bipyridines and [Bi(C_10_H_8_N_2_)(NO_3_)_4_]^-^coordination anions, connected through hydrogen bonds and π–π stacking interactions.

The formula unit of the title compound consists of one coordination anion, [Bi(C_10_H_8_N_2_)(NO_3_)_4_]^-^, which has a crystallographic twofold axis symmetry, and one disordered monoprotonated 2,2-bipyridine (Fig. 1). The disordered protonated 2,2-bipyridine in the structure of title compound is similar to that found in the compound (C_10_H_9_N_2_)(BiI_4_). In the coordination anion, the Bi(III) center is coordinated by two nitrogen atoms from one C_10_H_8_N_2_ ligand and eight oxygen atoms from four NO_3_^-^ anions with two Bi—N equal distances of 2.444 (5) Å and Bi—O distances ranging from 2.470 (6) to 2.703 (8) Å. On the basis of bond strength calculations, the bond valence sum (BVS) for Bi1 is 3.30 which suggests that Bi has the +3 oxidation state.

Besides electrostatic interactions, the coordination anions [Bi(C_10_H_8_N_2_)(NO_3_)_4_]^-^and protonated 2,2-bipyridines are connected *via* N—H···O hydrogen bonds [N4—H7···O4, 3.145 (10) Å, 149°] into chains. These chains are further linked into two-dimensional layered structure through the π–π stacking interactions between bipy ligands along the *c* axis (Fig. 2). Interacting aromatic rings of bipy ligands in these stacks are parallel to each other with the interplanar distance of 2.868 (4) Å.

### Experimental

All chemicals were of reagent grade quality obtained from commercial sources and used without further purification. The 10 ml portions of ethanol solutions of C_10_H_8_N_2_ (0.16 g, 1 mmol) and 2,6-pyridinedicarboxylic acid (1 mmol, 0.17 g) were mixed, then 12 ml of ethanol solution of Bi(NO_3_)_3_.5H_2_O (0.49 g, 1 mmol) was added. After stirring for half an hour, the solution was filtered and left for slowly evaporation at room temperature to obtain colorless block crystals suitable for X-ray structure determination. Yield: 70% (based on Bi). Anal. calcd. (%) for C_20_H_17_BiN_8_O_12_: C, 31.18; H, 2.22; N, 14.55. Found (%): C, 31.26; H, 2.09; N, 14.71.

### Refinement

The H8 and H10 atoms were located from Fourier difference maps and refined with distance restraints of 0.93 Å, other H atoms were positioned geometrically and refined using a riding model, with C—H = 0.93 Å and with *U*_iso_(H) = 1.2 times *U*_eq_(C). The occupancy for C7/N4 and C11/N5 sites is 50% C and 50% N. In addition, the O1, N1 and O1, Bi1 atoms were refined with restraints (delu 0.01, isor 0.001 for O1).

### Crystal data

**Table:**

(C_10_H_9_N_2_)[Bi(NO_3_)_4_(C_10_H_8_N_2_)]	*F*(000) = 1488
*M**_r_* = 770.40	*D*_x_ = 2.049 Mg m^−^^3^
Monoclinic, *C*2/*c*	Mo *K*α radiation, λ = 0.71073 Å
Hall symbol: -C 2yc	Cell parameters from 2470 reflections
*a* = 14.711 (5) Å	θ = 2.4–22.5°
*b* = 10.169 (3) Å	µ = 7.14 mm^−^^1^
*c* = 16.832 (5) Å	*T* = 293 K
β = 97.275 (6)°	Block, colorless
*V* = 2497.7 (13) Å^3^	0.26 × 0.24 × 0.18 mm
*Z* = 4	

### Data collection

**Table:**

Bruker APEXII CCD diffractometer	2224 independent reflections
Radiation source: fine-focus sealed tube	1895 reflections with *I* > 2σ(*I*)
graphite	*R*_int_ = 0.036
φ and ω scans	θ_max_ = 25.1°, θ_min_ = 2.4°
Absorption correction: multi-scan (*SADABS*; Bruker, 2008)	*h* = −13→17
*T*_min_ = 0.258, *T*_max_ = 0.360	*k* = −12→12
5998 measured reflections	*l* = −20→17

### Refinement

**Table:**

Refinement on *F*^2^	Primary atom site location: structure-invariant direct methods
Least-squares matrix: full	Secondary atom site location: difference Fourier map
*R*[*F*^2^ > 2σ(*F*^2^)] = 0.035	Hydrogen site location: inferred from neighbouring sites
*wR*(*F*^2^) = 0.084	H atoms treated by a mixture of independent and constrained refinement
*S* = 1.02	*w* = 1/[σ^2^(*F*_o_^2^) + (0.0459*P*)^2^ + 9.2649*P*] where *P* = (*F*_o_^2^ + 2*F*_c_^2^)/3
2224 reflections	(Δ/σ)_max_ < 0.001
194 parameters	Δρ_max_ = 1.13 e Å^−^^3^
11 restraints	Δρ_min_ = −1.43 e Å^−^^3^

### Special details

**Table:**

Geometry. All s.u.'s (except the s.u. in the dihedral angle between two l.s. planes) are estimated using the full covariance matrix. The cell s.u.'s are taken into account individually in the estimation of s.u.'s in distances, angles and torsion angles; correlations between s.u.'s in cell parameters are only used when they are defined by crystal symmetry. An approximate (isotropic) treatment of cell s.u.'s is used for estimating s.u.'s involving l.s. planes.

### Fractional atomic coordinates and isotropic or equivalent isotropic displacement parameters (Å^2^)

**Table:**

	*x*	*y*	*z*	*U*_iso_*/*U*_eq_	Occ. (<1)
Bi1	0.0000	0.43500 (3)	0.2500	0.03845 (15)	
C1	−0.1200 (5)	0.6312 (8)	0.3579 (4)	0.0481 (18)	
H1	−0.1338	0.5504	0.3794	0.058*	
C2	−0.1652 (5)	0.7417 (7)	0.3805 (4)	0.0487 (18)	
H2	−0.2072	0.7358	0.4173	0.058*	
C3	−0.1466 (5)	0.8595 (7)	0.3475 (4)	0.0508 (19)	
H3	−0.1785	0.9348	0.3589	0.061*	
C4	−0.0802 (5)	0.8654 (7)	0.2972 (4)	0.0437 (17)	
H4	−0.0650	0.9462	0.2767	0.052*	
C5	−0.0351 (4)	0.7518 (6)	0.2764 (4)	0.0335 (14)	
C6	0.0361 (5)	0.0371 (7)	−0.0162 (4)	0.0473 (18)	
C7	0.0484 (6)	0.1654 (8)	0.0057 (4)	0.063 (2)	0.50
H4A	0.0131	0.2045	0.0413	0.075*	0.25
H7	0.0132	0.2045	0.0413	0.075*	0.50
C8	0.1149 (10)	0.2352 (12)	−0.0267 (8)	0.094 (4)	
H8	0.100 (6)	0.315 (5)	−0.006 (5)	0.07 (3)*	
C9	0.1650 (8)	0.179 (2)	−0.0789 (8)	0.111 (5)	
H9	0.2086	0.2271	−0.1017	0.134*	
C10	0.1511 (9)	0.0488 (18)	−0.0982 (7)	0.098 (4)	
H10	0.162 (10)	0.000 (13)	−0.143 (6)	0.15 (6)*	
C11	0.0859 (6)	−0.0230 (9)	−0.0674 (5)	0.067 (2)	0.50
H5	0.0760	−0.1109	−0.0813	0.080*	0.25
H11	0.0760	−0.1109	−0.0813	0.080*	0.50
O1	−0.1017 (5)	0.2143 (8)	0.2235 (4)	0.095 (2)	
O2	−0.1374 (4)	0.3450 (6)	0.3126 (4)	0.0784 (19)	
O3	−0.2044 (5)	0.1589 (7)	0.2956 (5)	0.101 (2)	
O4	−0.0397 (5)	0.3946 (7)	0.0954 (4)	0.0789 (18)	
O5	−0.1352 (4)	0.5005 (6)	0.1561 (3)	0.0635 (15)	
O6	−0.1767 (5)	0.4407 (7)	0.0349 (4)	0.094 (2)	
N1	−0.1488 (5)	0.2376 (7)	0.2795 (5)	0.0631 (19)	
N2	−0.1182 (5)	0.4426 (7)	0.0941 (4)	0.0593 (18)	
N3	−0.0577 (4)	0.6348 (5)	0.3070 (3)	0.0365 (12)	
N4	0.0484 (6)	0.1654 (8)	0.0057 (4)	0.063 (2)	0.50
N5	0.0859 (6)	−0.0230 (9)	−0.0674 (5)	0.067 (2)	0.50

### Atomic displacement parameters (Å^2^)

**Table:**

	*U*^11^	*U*^22^	*U*^33^	*U*^12^	*U*^13^	*U*^23^
Bi1	0.0478 (2)	0.0270 (2)	0.0448 (2)	0.000	0.02219 (16)	0.000
C1	0.055 (5)	0.047 (4)	0.047 (4)	−0.004 (4)	0.023 (4)	0.000 (4)
C2	0.052 (4)	0.052 (5)	0.046 (4)	0.010 (3)	0.022 (3)	−0.005 (4)
C3	0.061 (5)	0.042 (4)	0.053 (5)	0.017 (4)	0.017 (4)	−0.006 (4)
C4	0.063 (5)	0.026 (4)	0.040 (4)	0.009 (3)	0.002 (3)	0.002 (3)
C5	0.041 (4)	0.029 (3)	0.032 (3)	0.002 (3)	0.012 (3)	−0.005 (3)
C6	0.047 (4)	0.053 (5)	0.040 (4)	0.011 (3)	0.000 (3)	−0.002 (3)
C7	0.076 (5)	0.053 (5)	0.059 (5)	0.000 (4)	0.006 (4)	−0.004 (4)
C8	0.116 (10)	0.066 (8)	0.093 (8)	−0.020 (7)	−0.018 (8)	0.003 (7)
C9	0.062 (7)	0.181 (15)	0.089 (9)	−0.025 (9)	−0.001 (6)	0.065 (10)
C10	0.070 (7)	0.164 (14)	0.062 (7)	0.047 (9)	0.012 (6)	0.023 (9)
C11	0.069 (5)	0.080 (6)	0.052 (4)	0.026 (4)	0.011 (4)	−0.002 (4)
O1	0.096 (2)	0.094 (2)	0.097 (2)	−0.0016 (10)	0.0153 (10)	−0.0016 (10)
O2	0.092 (5)	0.055 (4)	0.097 (5)	−0.028 (3)	0.048 (4)	−0.016 (3)
O3	0.093 (5)	0.064 (4)	0.152 (7)	−0.031 (4)	0.039 (5)	0.020 (4)
O4	0.085 (5)	0.087 (5)	0.071 (4)	−0.001 (4)	0.034 (4)	−0.023 (3)
O5	0.064 (4)	0.073 (4)	0.054 (3)	0.006 (3)	0.008 (3)	−0.015 (3)
O6	0.103 (5)	0.121 (6)	0.053 (4)	−0.031 (4)	−0.009 (4)	−0.013 (4)
N1	0.062 (4)	0.046 (4)	0.087 (5)	−0.007 (3)	0.031 (4)	0.017 (4)
N2	0.071 (5)	0.058 (4)	0.053 (4)	−0.013 (4)	0.021 (4)	−0.010 (4)
N3	0.046 (3)	0.027 (3)	0.039 (3)	0.004 (2)	0.013 (3)	0.001 (2)
N4	0.076 (5)	0.053 (5)	0.059 (5)	0.000 (4)	0.006 (4)	−0.004 (4)
N5	0.069 (5)	0.080 (6)	0.052 (4)	0.026 (4)	0.011 (4)	−0.002 (4)

### Geometric parameters (Å, °)

**Table:**

Bi1—N3	2.444 (5)	C6—C11	1.346 (10)
Bi1—N3^i^	2.444 (5)	C6—C7	1.362 (10)
Bi1—O5^i^	2.470 (6)	C6—C6^ii^	1.463 (15)
Bi1—O5	2.470 (6)	C7—C8	1.376 (15)
Bi1—O2	2.564 (6)	C7—H4A	0.9300
Bi1—O2^i^	2.564 (6)	C7—H7	0.9300
Bi1—O4	2.626 (6)	C8—C9	1.345 (19)
Bi1—O4^i^	2.626 (6)	C8—H8	0.92 (2)
Bi1—O1	2.703 (8)	C9—C10	1.368 (19)
Bi1—O1^i^	2.703 (8)	C9—H9	0.9300
C1—N3	1.333 (8)	C10—C11	1.359 (17)
C1—C2	1.383 (10)	C10—H10	0.93 (2)
C1—H1	0.9300	C11—H5	0.9300
C2—C3	1.363 (10)	C11—H11	0.9299
C2—H2	0.9300	O1—N1	1.261 (9)
C3—C4	1.372 (10)	O2—N1	1.228 (8)
C3—H3	0.9300	O3—N1	1.200 (8)
C4—C5	1.399 (9)	O4—N2	1.252 (9)
C4—H4	0.9300	O5—N2	1.250 (8)
C5—N3	1.353 (8)	O6—N2	1.232 (10)
C5—C5^i^	1.445 (12)		
			
N3—Bi1—N3^i^	67.5 (2)	C3—C2—H2	120.8
N3—Bi1—O5^i^	79.4 (2)	C1—C2—H2	120.8
N3^i^—Bi1—O5^i^	74.64 (18)	C2—C3—C4	119.0 (6)
N3—Bi1—O5	74.64 (18)	C2—C3—H3	120.5
N3^i^—Bi1—O5	79.4 (2)	C4—C3—H3	120.5
O5^i^—Bi1—O5	148.7 (3)	C3—C4—C5	121.1 (7)
N3—Bi1—O2	78.73 (19)	C3—C4—H4	119.5
N3^i^—Bi1—O2	142.1 (2)	C5—C4—H4	119.5
O5^i^—Bi1—O2	116.5 (2)	N3—C5—C4	118.9 (6)
O5—Bi1—O2	75.3 (2)	N3—C5—C5^i^	117.6 (3)
N3—Bi1—O2^i^	142.1 (2)	C4—C5—C5^i^	123.5 (4)
N3^i^—Bi1—O2^i^	78.73 (19)	C11—C6—C7	122.9 (8)
O5^i^—Bi1—O2^i^	75.3 (2)	C11—C6—C6^ii^	119.0 (9)
O5—Bi1—O2^i^	116.5 (2)	C7—C6—C6^ii^	118.1 (8)
O2—Bi1—O2^i^	138.2 (3)	C6—C7—C8	117.7 (9)
N3—Bi1—O4	118.3 (2)	C6—C7—H4A	121.2
N3^i^—Bi1—O4	77.65 (19)	C8—C7—H4A	121.2
O5^i^—Bi1—O4	137.4 (2)	C6—C7—H7	121.2
O5—Bi1—O4	49.3 (2)	C8—C7—H7	121.2
O2—Bi1—O4	105.3 (2)	H4A—C7—H7	0.0
O2^i^—Bi1—O4	68.0 (2)	C9—C8—C7	120.8 (11)
N3—Bi1—O4^i^	77.65 (19)	C9—C8—H8	143 (6)
N3^i^—Bi1—O4^i^	118.3 (2)	C7—C8—H8	95 (6)
O5^i^—Bi1—O4^i^	49.3 (2)	C8—C9—C10	119.4 (11)
O5—Bi1—O4^i^	137.4 (2)	C8—C9—H9	120.3
O2—Bi1—O4^i^	68.0 (2)	C10—C9—H9	120.3
O2^i^—Bi1—O4^i^	105.3 (2)	C11—C10—C9	121.3 (11)
O4—Bi1—O4^i^	162.0 (3)	C11—C10—H10	103 (10)
N3—Bi1—O1	122.9 (2)	C9—C10—H10	132 (10)
N3^i^—Bi1—O1	147.0 (2)	C6—C11—C10	117.8 (10)
O5^i^—Bi1—O1	135.5 (2)	C6—C11—H5	121.1
O5—Bi1—O1	74.6 (2)	C10—C11—H5	121.1
O2—Bi1—O1	47.4 (2)	C6—C11—H11	121.1
O2^i^—Bi1—O1	94.8 (2)	C10—C11—H11	121.1
O4—Bi1—O1	70.0 (2)	H5—C11—H11	0.1
O4^i^—Bi1—O1	94.7 (2)	N1—O1—Bi1	93.8 (5)
N3—Bi1—O1^i^	147.0 (2)	N1—O2—Bi1	101.6 (5)
N3^i^—Bi1—O1^i^	122.9 (2)	N2—O4—Bi1	92.6 (4)
O5^i^—Bi1—O1^i^	74.6 (2)	N2—O5—Bi1	100.2 (5)
O5—Bi1—O1^i^	135.5 (2)	O3—N1—O2	123.3 (8)
O2—Bi1—O1^i^	94.8 (2)	O3—N1—O1	119.9 (8)
O2^i^—Bi1—O1^i^	47.4 (2)	O2—N1—O1	116.7 (7)
O4—Bi1—O1^i^	94.7 (2)	O6—N2—O5	119.3 (8)
O4^i^—Bi1—O1^i^	70.0 (2)	O6—N2—O4	123.8 (7)
O1—Bi1—O1^i^	67.7 (3)	O5—N2—O4	116.8 (7)
N3—C1—C2	123.2 (7)	C1—N3—C5	119.4 (6)
N3—C1—H1	118.4	C1—N3—Bi1	122.0 (5)
C2—C1—H1	118.4	C5—N3—Bi1	117.8 (4)
C3—C2—C1	118.3 (6)		
			
N3—C1—C2—C3	1.6 (12)	N3^i^—Bi1—O5—N2	88.9 (5)
C1—C2—C3—C4	−3.9 (11)	O5^i^—Bi1—O5—N2	123.2 (5)
C2—C3—C4—C5	3.3 (11)	O2—Bi1—O5—N2	−119.6 (5)
C3—C4—C5—N3	−0.2 (10)	O2^i^—Bi1—O5—N2	17.3 (5)
C3—C4—C5—C5^i^	179.7 (8)	O4—Bi1—O5—N2	6.1 (4)
C11—C6—C7—C8	0.1 (12)	O4^i^—Bi1—O5—N2	−150.5 (4)
C6^ii^—C6—C7—C8	−178.4 (9)	O1—Bi1—O5—N2	−70.5 (5)
C6—C7—C8—C9	0.8 (15)	O1^i^—Bi1—O5—N2	−37.8 (6)
C7—C8—C9—C10	−1.8 (18)	Bi1—O2—N1—O3	175.6 (7)
C8—C9—C10—C11	2.0 (17)	Bi1—O2—N1—O1	−7.8 (8)
C7—C6—C11—C10	0.0 (12)	Bi1—O1—N1—O3	−176.0 (7)
C6^ii^—C6—C11—C10	178.5 (9)	Bi1—O1—N1—O2	7.3 (8)
C9—C10—C11—C6	−1.0 (15)	Bi1—O5—N2—O6	172.3 (6)
N3—Bi1—O1—N1	−28.6 (6)	Bi1—O5—N2—O4	−11.0 (8)
N3^i^—Bi1—O1—N1	−127.7 (5)	Bi1—O4—N2—O6	−173.2 (7)
O5^i^—Bi1—O1—N1	81.7 (6)	Bi1—O4—N2—O5	10.2 (7)
O5—Bi1—O1—N1	−88.2 (5)	C2—C1—N3—C5	1.5 (11)
O2—Bi1—O1—N1	−4.2 (5)	C2—C1—N3—Bi1	−168.7 (6)
O2^i^—Bi1—O1—N1	155.6 (5)	C4—C5—N3—C1	−2.2 (10)
O4—Bi1—O1—N1	−140.0 (5)	C5^i^—C5—N3—C1	177.9 (7)
O4^i^—Bi1—O1—N1	49.8 (5)	C4—C5—N3—Bi1	168.4 (5)
O1^i^—Bi1—O1—N1	115.9 (6)	C5^i^—C5—N3—Bi1	−11.5 (9)
N3—Bi1—O2—N1	163.7 (6)	N3^i^—Bi1—N3—C1	174.4 (7)
N3^i^—Bi1—O2—N1	136.6 (5)	O5^i^—Bi1—N3—C1	−107.9 (5)
O5^i^—Bi1—O2—N1	−124.2 (5)	O5—Bi1—N3—C1	89.8 (6)
O5—Bi1—O2—N1	86.8 (5)	O2—Bi1—N3—C1	12.1 (5)
O2^i^—Bi1—O2—N1	−26.7 (5)	O2^i^—Bi1—N3—C1	−156.7 (5)
O4—Bi1—O2—N1	47.2 (6)	O4—Bi1—N3—C1	113.5 (5)
O4^i^—Bi1—O2—N1	−115.2 (6)	O4^i^—Bi1—N3—C1	−57.6 (5)
O1—Bi1—O2—N1	4.4 (5)	O1—Bi1—N3—C1	30.1 (6)
O1^i^—Bi1—O2—N1	−49.0 (6)	O1^i^—Bi1—N3—C1	−69.4 (7)
N3—Bi1—O4—N2	−36.8 (5)	N3^i^—Bi1—N3—C5	4.1 (3)
N3^i^—Bi1—O4—N2	−92.6 (5)	O5^i^—Bi1—N3—C5	81.7 (5)
O5^i^—Bi1—O4—N2	−142.9 (4)	O5—Bi1—N3—C5	−80.6 (5)
O5—Bi1—O4—N2	−6.0 (4)	O2—Bi1—N3—C5	−158.3 (5)
O2—Bi1—O4—N2	48.4 (5)	O2^i^—Bi1—N3—C5	33.0 (6)
O2^i^—Bi1—O4—N2	−175.3 (5)	O4—Bi1—N3—C5	−56.9 (5)
O4^i^—Bi1—O4—N2	113.7 (5)	O4^i^—Bi1—N3—C5	132.1 (5)
O1—Bi1—O4—N2	80.5 (5)	O1—Bi1—N3—C5	−140.3 (5)
O1^i^—Bi1—O4—N2	144.7 (5)	O1^i^—Bi1—N3—C5	120.3 (5)
N3—Bi1—O5—N2	158.3 (5)		

Symmetry codes: (i) −*x*, *y*, −*z*+1/2; (ii) −*x*, −*y*, −*z*.

### Hydrogen-bond geometry (Å, °)

**Table:**

*D*—H···*A*	*D*—H	H···*A*	*D*···*A*	*D*—H···*A*
N4—H7···O4	0.93	2.31	3.145 (10)	149
